# Intermittent Theta Burst Stimulation of the Prefrontal Cortex in Cocaine Use Disorder: A Pilot Study

**DOI:** 10.3389/fnins.2019.00765

**Published:** 2019-07-25

**Authors:** Angela Sanna, Liana Fattore, Paola Badas, Giorgio Corona, Viola Cocco, Marco Diana

**Affiliations:** ^1^Department of Medical Science and Public Health, Section of Neurology, University of Cagliari, Cagliari, Italy; ^2^CNR Institute of Neuroscience-Cagliari, National Research Council, Cagliari, Italy; ^3^rTMS Italia, Cagliari, Italy; ^4^“G. Minardi” Laboratory of Cognitive Neuroscience, Department of Chemistry and Pharmacy, University of Sassari, Sassari, Italy

**Keywords:** cocaine, transcranial magnetic stimulation, intermittent theta burst stimulation, craving, neuromodulation

## Abstract

Transcranial Magnetic Stimulation (TMS) is earning a role in the therapeutic arsenal of cocaine use disorder (CUD). A widespread and still growing number of studies have reported beneficial use of repeated TMS (rTMS) in reduction of craving, intake and cue-induced craving in cocaine addicts. In spite of these encouraging findings, many issues are still unresolved such as brain area to be stimulated, laterality of the effects, coil geometry and stimulation protocols/parameters. Intermittent theta burst stimulation (iTBS) is a more tolerable protocol administered at lower intensities and shorter intervals than conventional rTMS protocols. Yet, its effects on cocaine craving and length of abstinence in comparison with standard high frequency (10–15 Hz) protocols have never been evaluated so far. In the present paper, we describe the effect of the bilateral iTBS of the prefrontal cortex (PFC) in a population (*n* = 25) of treatment-seeking cocaine addicts, in an outpatient setting, and compare them with 15 Hz stimulation of the same brain area (*n* = 22). The results indicate that iTBS produces effects on cocaine consumption and cocaine craving virtually superimposable to the 15 Hz rTMS group. Both treatments had low numbers of dropouts and similar side-effects, safety and tolerability profiles. While larger studies are warranted to confirm these observations, iTBS appears to be a valid approach to be considered in treatment-seeking cocaine addicts, especially in light of its brief duration (3 min) vs. 15 Hz stimulation (15 min). The use of iTBS would allow increasing the number of patients treated per day with current rTMS devices, thus reducing patient discomfort and hopefully reducing drop-out rates without compromising clinical effectiveness.

## Introduction

According to the World Drug Report 2017, 17 million people were past-year cocaine users in 2015 and cocaine seizures were reported in 153 countries during the period 2010–2015 (United Nations Office on Drugs and Crime [UNODC], 2017), suggesting that trafficking in cocaine is a global phenomenon. Noteworthy, after cannabis, cocaine accounts for the largest quantities seized. After a long-term decline, coca bush cultivation increased over the period 2013–2015 and current data on drug production, trafficking and consumption confirm an extension of the market for cocaine (United Nations Office on Drugs and Crime [UNODC], 2017). In recent years, a wealth of clinical and animal studies has advanced our understanding of the brain mechanisms sustaining cocaine use and promoting dependence (Hanlon and Canterberry, 2012; Castilla-Ortega et al., 2017; Dobbs et al., 2017). Numerous efforts are being made to target novel molecular and cellular targets and effective strategies against cocaine addiction (Blum et al., 2009; Penberthy et al., 2010; Davidson, 2016). To date, though, pharmacological and psychological therapies for treating cocaine dependence have shown only limited success. Hence, research is very active and different experimental approaches are currently under investigation.

Cocaine use alters several brain processes of the addiction cycle, from reinforcement learning to inhibitory control (Koob and Volkow, 2010; Everitt, 2014). The prefrontal cortex (PFC) is strongly involved in cognitive impairment induced by chronic cocaine use and represents a crucial brain area where cognitive mechanisms are thought to be generated. Clinical evidence links PFC hypo-functionality to the loss of inhibitory control over drug seeking (Goldstein and Volkow, 2011). Among others, neuroimaging studies have greatly contributed to disentangle the neural circuits that become dysfunctional after repeated use of cocaine and contribute to the development of dependence. Chronic cocaine users typically present significant alterations and dysfunctions in the PFC, including cortical hypoactivity (Kaufman et al., 2003), brain volume reduction (Moreno-López et al., 2012), impaired executive functions and dysregulated neurotransmitters systems (Volkow et al., 2003; Licata and Renshaw, 2010). Cocaine users were also consistently found to have lower baseline cortical excitability, i.e., higher motor thresholds, than non-cocaine users (Boutros et al., 2001; Sundaresan et al., 2007; Gjini et al., 2012). A tonically stable hypofunctioning dopaminergic state (Melis et al., 2005) and a general malfunctioning of the PFC (Nogueira et al., 2006), accompanied by increased salience attribution to the drug (Volkow et al., 2005), have been postulated to promote compulsive cocaine intake. As for other drugs of abuse, persistent compulsive use of cocaine is hypothesized to be maintained by enduring changes induced by the drug in specific forebrain circuits that are involved in affective (e.g., ventral striatum) and cognitive (e.g., PFC) mechanisms (Fattore and Diana, 2016). Specifically, a progressive decrease in PFC control over drug-seeking and drug-taking behavior along with a shift from cortical to dorsal striatal control of behavior has been postulated to be at the basis of the transition from intentional to habitual and progressively compulsive drug use (Everitt and Robbins, 2013). Further, *in vivo* optogenetic and electrical stimulation of the infralimbic cortex (a portion of the cingulate cortex) significantly prevents compulsive cocaine seeking in animals (Chen et al., 2013), supporting the hypothesis that stimulation of PFC could mitigate cocaine seeking and consumption. Intriguingly, the modulation of prefrontal cortical areas through rTMS has been shown to be beneficial in reducing cocaine intake and alleviating some drug-related aspects in cocaine addicts (Bolloni et al., 2016). Indeed, generation of an electromagnetic field able to cross painlessly the skull and to influence the brain matter appears a promising approach to cocaine use disorder (CUD) and other substances use disorders, especially in light of its minimal side-effects.

Transcranial magnetic stimulation (TMS) has been reported to be useful in the treatment of several neuropsychiatric diseases (Daskalakis et al., 2002; Kobayashi and Pascual-Leone, 2003; Lefaucheur et al., 2014). A single TMS pulse lasts a few milliseconds, but when pulses are applied repetitively, as in the repetitive TMS protocol (rTMS), they can modulate long-term cortical excitability and affect the function of neuronal circuits in a frequency-dependent manner (Eldaief et al., 2011). Specifically, through electromagnetic induction rTMS at a low frequency (1 Hz) is typically considered to have inhibitory effects and induce long-term depression (LTD)-like changes (Chen et al., 1997) while rTMS at a high-frequency (from 5 Hz upward) is excitatory and can induce long-term potentiation (LTP)-like effects (Pascual-Leone et al., 1994). In rats, acute high-frequency (20 Hz) rTMS of the frontal cortex was shown to induce dopamine release in several parts of the brain reward system, including the dorsal hippocampus and the nucleus accumbens (Keck et al., 2002). In humans, high frequency rTMS of the human PFC was reported to stimulate dopamine release in the ipsilateral caudate nucleus (Strafella et al., 2001) and to modulate dopamine release in the ipsilateral anterior cingulate and orbitofrontal cortices (Cho and Strafella, 2009). Moreover, in alcohol dependent patients, rTMS significantly reduced blood cortisol levels and decreased prolactinemia, which suggests an increase in dopamine levels (Ceccanti et al., 2015). In light of its modulatory effect on both the mesolimbic and the mesostriatal dopaminergic systems, high frequency rTMS of frontal brain regions has been proposed to be useful in neuropsychiatry disorders associated with subcortical dopamine dysfunction such as alcohol and drug addiction in general (Diana, 2011).

Drug craving has been acknowledged as a relevant construct in the pathophysiology of addiction. As such, it has been included by the DSM-V in the list of crucial clinical symptom of substance use disorders and has been proposed to be routinely included as a clinical outcome in research on treatments for substance-use disorders (Tiffany and Wray, 2012). Patients seeking treatment for cocaine dependence often identify amplified craving as a major trigger of drug relapses. High frequency (15 Hz) rTMS-for several days-to the left dorsolateral prefrontal cortex (dlPFC) using “figure-of-eight” coil was shown to reduce cocaine craving and intake as compared with a standard pharmacological treatment (Terraneo et al., 2016). Further, a single session of high frequency (10 Hz) rTMS transiently reduced cocaine craving when applied to the right dlPFC but not the left dlPFC (Camprodon et al., 2007), although discrepant results were also reported (Politi et al., 2008). In fact, high frequency rTMS targeting the left dlPFC was reported to not affect craving but to gradually reduce cocaine consumption over 10 daily sessions (Politi et al., 2008). Other rTMS protocols and types of coils are currently under evaluation for their ability to reduce craving and cocaine intake in addicted patients. Some authors, for example, tested 20 Hz deep TMS using a Hesed (H)-coil that stimulates bilaterally and simultaneously the area of interest and observed a reduction of cocaine craving in patients with CUD after a month of treatment (Rapinesi et al., 2016). Using the same type of coil, 10 Hz rTMS was also shown to improve abstinence from cocaine in patients with CUD at 3 and 6 months, but not at 1 month, i.e., immediately after the treatment (Bolloni et al., 2016), thereby suggesting a lasting and prolonged effect. More recently, Martinez and colleagues demonstrated that 10 Hz rTMS delivered with the newly designed H7 coil, which allows to target the medial prefrontal cortex (mPFC) and the dorsal anterior cingulate, reduces cocaine self-administration in volunteers with CUD (Martinez et al., 2018).

The insular cortex, with its important connections with the orbitofrontal and the anterior cingulate cortices, the thalamus, amygdala and globus pallidus, is another interesting brain target for TMS that showed to be promising in treating drug addiction. By using a peculiar H-shaped deep coil that allows rTMS to target deeper brain structures like the insula (Roth et al., 2002) it was observed, for example, that 10 Hz deep rTMS of the insular and prefrontal cortices significantly decrease the number of cigarettes smoked in nicotine dependent heavy smokers (Dinur-Klein et al., 2014). Given the crucial role of the insula in incentive motivational processes (Naqvi and Bechara, 2010; Naqvi et al., 2014) and in the contextual control over cocaine-seeking (Arguello et al., 2017) and cue-induced reinstatement (Cosme et al., 2015), modulating the function of both the prefrontal and insular cortices could result in a novel therapeutic strategy to be extended to cocaine addicts. In partial support to this hypothesis, deep rTMS of the insular cortex was recently reported to significantly modulate dopamine release in healthy subjects (Malik et al., 2018).

Theta burst stimulation (TBS), is a patterned form of TMS that can be applied using continuous or intermittent protocols (Huang et al., 2005; Di Lazzaro et al., 2008) that is widely believed to represent cellular learning in a Hebbian form of long-term synaptic plasticity (Larson et al., 1986). Unlike high frequency rTMS, TBS mimics endogenous theta rhythms thus improving induction of synaptic LTP (Suppa et al., 2016). Like high frequency rTMS, when applied in an intermittent (LTP-like) and continuous (LTD-like) manner TBS induces, respectively, a potentiation and a depression of cortical excitability. Continuous theta burst stimulation (cTBS) to the left ventromedial prefrontal cortex (vmPFC) significantly attenuated cue-related functional connectivity between the left vmPFC and a number of other brain regions, including the left and right insula (Kearney-Ramos et al., 2018). In keeping with this, when tested as add-on treatment to cognitive-behavioral therapy, 4 sessions of iTBS were reported to reduce nicotine craving and improve long-term abstinence in smokers (Dieler et al., 2014). Noteworthy, iTBS gives short TBS trains intermittently and is administered at lower intensities and shorter intervals than conventional rTMS protocol, improving tolerability and safety in patients (Oberman et al., 2011). Moreover, application of iTBS requires 2–3 min vs. 15–30 min typically required by application of rTMS, making the iTBS protocol more acceptable to patients.

At present, no study has compared the effects of iTBS treatment sessions vs. standard high-frequency TMS protocol in cocaine-dependent patients. Thus, a key practical question remains unaddressed: does iTBS perform comparably to the existing standard of care in cocaine addicts? This study aims to evaluate the effectiveness of iTBS targeting the PFC and the insular cortex bilaterally on cocaine craving and intake and to compare safety and effectiveness for PFC-rTMS with 15 Hz (15 min) versus iTBS (3 min) protocols in cocaine addicts.

## Materials and Methods

The study was conducted at the rTMS Center in Milan, Italy. All participants provided written informed consent to participate in the study. The consent form included all information regarding the nature of the TMS treatment and possible side effects. The study endorsed the Principles of Human Rights, as adopted by the World Medical Association (18th WMA General Assembly) in 1964 in Helsinki (Finland) and then amended by the 64th WMA General Assembly in 2013 in Fortaleza (Brazil).

### Subjects

Patients were treatment-seeking outpatients in treatment for CUD diagnosed according to DSM-V criteria (American Psychiatric Association [APA], 2013) that were enrolled in the study from September 2017 to September 2018. Inclusion criteria were: age between 18 and 65 years, current CUD (i.e., have a positive urine drug screen for cocaine), motivation to stop intake, ability to understand and sign the informed consent. Exclusion criteria were: medical devices (pace-maker, metal implants, device for inflating), personal or family history of epilepsy, pregnancy (Rossi et al., 2009). Screening included medical history, physical and neurological examinations. Patients were asked about the weekly amount of cocaine consumed at baseline and at the end of the treatment; cocaine consumption was evaluated twice a week by means of a commercial urine drug screen test (Home Health Ltd., Hertfordshire, United Kingdom).

### Questionnaires

Craving for cocaine was assessed once a week using the cocaine craving questionnaire (CCQ-brief) (Tiffany et al., 1993), a 5 questions questionnaire with 0–9 score visual analogical scale (Weiss modified CCQ) shown to be a valid and reliable measure of cocaine craving that exhibits high internal reliability (Sussner et al., 2006). The patient’s risk for developing problems based on his/her use of cocaine was calculated at baseline and at the end of the treatment by means of the NIDA Modified WHO ASSIST questionnaire (*World Health Organization, WHO). Alcohol, Smoking and Substance Involvement Screening Test, Version 3.0*), a quick screening test for patients with a substance use disorder that the United States National Institute on Drug Abuse (NIDA) recommends as Resource Guide designed to assist clinicians screening adult patients for drug use was used to calculate. In this study 22 and 25 patients were enrolled for HF and TBS protocol, respectively.

### High Frequency (HF) and Intermittent Theta Burst Stimulation (iTBS)

**FIGURE 1:**
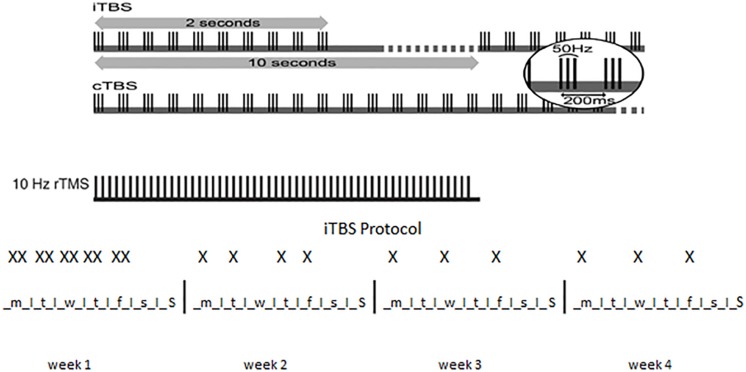
Patterns of TBS. *Top*: the basic element of TBS is a 3-pulse burst at 50 Hz delivered every 200 ms (i.e., 5 Hz). A train of 10 bursts lasting for 2 s is given every 10 s for 20 cycles in iTBS, for a total of 190 s (iTBS) while 100 or 200 continuous bursts are given continuously for 20 or 40 s, respectively, in cTBS. 10 Hz rTMS is illustrated for comparison. *Bottom*: X indicates a single iTBS session in the 4 weeks of treatment. Note that two sessions are administered in the same day during the 1st week of treatment.

Magstim Rapid stimulator (Magstim Company, Whitland, Wales, United Kingdom) was used along with H4-Coil (Brainsway Ltd., Jerusalem, Israel) (Zangen et al., 2005), specifically designed to stimulate bilateral PFC and insula symmetrically (Tendler et al., 2016). As illustrated in Figure 1, for both the high-frequency (HF) and the iTBS protocols, subjects received 20 stimulations over 4 weeks: 10 stimulations during the 1st week (2 daily sessions from Monday to Friday, with at least 1 h interval between the two sessions), 4 stimulations during the 2nd week (1 daily session for 4 days, Mon-Tue-Thu-Fri), 3 stimulations during the 3rd and 4th week (1 daily session for 3 alternate days).

For HF treatment the intensity of stimulation was set at 100% of resting motor threshold determined using visual observation of muscle twitch of the left hand. PFC was stimulated bilaterally at 15 Hz frequency, 40 trains of 60 pulses each (4 s) with 15 s inter-stimulus between trains for a total of 2400 pulses. ITBS was performed at 80% of active motor threshold determined using visual observation of muscle twitch of the left hand after a voluntary contraction. ITBS protocol consisted of bursts containing 3 pulses at 50 Hz repeated at 200-ms intervals for 2 s (i.e., at 5 Hz). A 2-second train of iTBS was repeated every 10 s for 190 s and 600 pulses (Huang et al., 2005).

### Statistical Analysis

In order to compare demographic features, multiple independent samples Student’s *t*-test and Chi Squared test was used for normally distributed variables and for categorical variables, respectively. Two-way Analysis of Variance (ANOVA) was used to evaluate the effect of the two TMS protocols on cocaine intake, craving scale and WHO ASSIST questionnaire between and within groups. Kaplan Meier curves were used to plot the cumulated proportion of event free patients in the groups and Log Rank was used to test the significance between pairs of curves.

## Results

Before starting the study, no statistical differences were found in the characteristics of the patients between the two groups at baseline (Table 1). Two patients in each group dropped out within the first 2 weeks of treatment.

**TABLE 1:** Baseline socio- demographic characteristics of the sample and clinical variables (M ± SD and range).

		**HF group (*n* = 22)**	**TBS group (*n* = 25)**	**Difference**
Gender (F/M)		1/22	1/25	
Age (years)		35,9 (8,5)	38,9 (8,0)	NS
Duration of cocaine use (years)		12,6 (8,0)	16,1 (9,2)	NS
Weekly cocaine amount (g)		9,6 (8,2)	8,1 (6,9)	NS
Route of administration	Inhalation	13	19	NS
	Smoke	7	6	NS
	Injective	2	0	NS
Psychiatric comorbidities		7/22	7/25	NS
	Mood disorder	4/22	4/25	NS
	Personality disorder	3/22	1/25	NS
	Anxiety	3/22	3/25	NS
Psychoactive prescription drugs		8/22	10/25	NS
	Mood stabilizers	2/22	1/25	NS
	Benzodiazepines	4/22	4/25	NS
	Antidepressants	3/22	3/25	NS
	Antipsychotics	2/22	3/25	NS
Other actual addiction		12/22	12/25	NS
	Nicotine	12/22	12/25	NS
	Alcohol	3/22	5/25	NS
	GAP	1/22	3/25	NS
	Heroin	2/22	2/25	NS
Past quit attempts		15/22	19/25	NS
Drop out		2/22	2/25	NS

As shown in Table 2, a few participants in both the 15 Hz rTMS and the iTBS group reported mild discomfort at the start of stimulation, especially during the first session, but overall there were no significant differences across groups. Both treatments were safe and there were no serious or unexpected adverse events related to the treatments.

**TABLE 2:** Side effects of HF and TBS protocols.

**Symptom**	**HF group (*n* = 22)**	**TBS group (*n* = 25)**	**Difference**
Headache	2/22	3/25	NS
Dizziness	1/22	1/25	NS
Sleepiness	4/22	4/25	NS
Insomnia	0/22	1/25	NS

### Effects on Cocaine Intake

**FIGURE 2:**
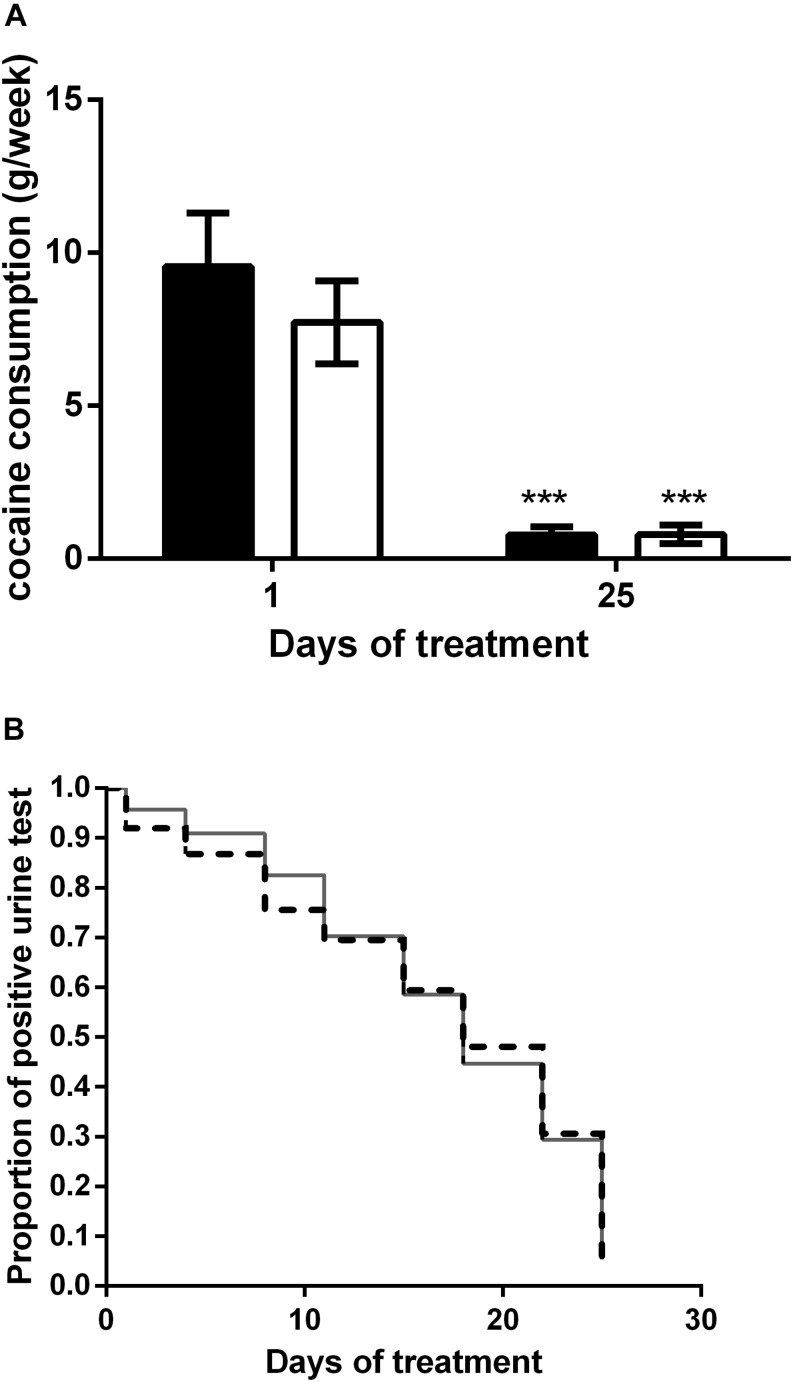
**(A)** Effect of HF (*black columns*) and iTBS (*white columns*) treatment on cocaine consumption (g/week). Data are presented as mean ± SEM. ^∗∗∗^*P* < 0.001 vs. day 1. **(B)** Kaplan Meier curve comparison between HF (*dashed line*) and iTBS (*solid line*). Data are presented as proportion of positive tests for each time point.

The effect of rTMS on cocaine consumption was evaluated twice/week by means of a urine test and by asking the patient about the quantitative of cocaine consumed every week. Figure 2A shows cocaine intake during the rTMS (HF, black bars) and the intermittent TBS (iTBS, white bars) treatments. With respect to the baseline value, both treatments significantly reduce the intake of cocaine with no statistical differences between the two groups. Indeed, two-way ANOVA revealed a significant effect of time (*F*_1,90_ = 49.97; *P* < 0.0001) but not of treatment (*F*_1,90_ = 0.67) or time × treatment interaction (*F*_1,90_ = 0.66). Figure 2B shows the Kaplan Meyer curves displaying the proportion of positive urine tests during the high-frequency rTMS (HF, dashed line) and the iTBS (solid line) treatments. The analysis revealed a significant decrease of positive tests at the end of the treatment with no difference between protocols (log rank *P* > 0.05). At the end of the treatment, 80 and 82% of the patients who underwent the HF and iTBS protocol, respectively, were found negative at the urine test.

### Effects on Craving and Risk

**FIGURE 3:**
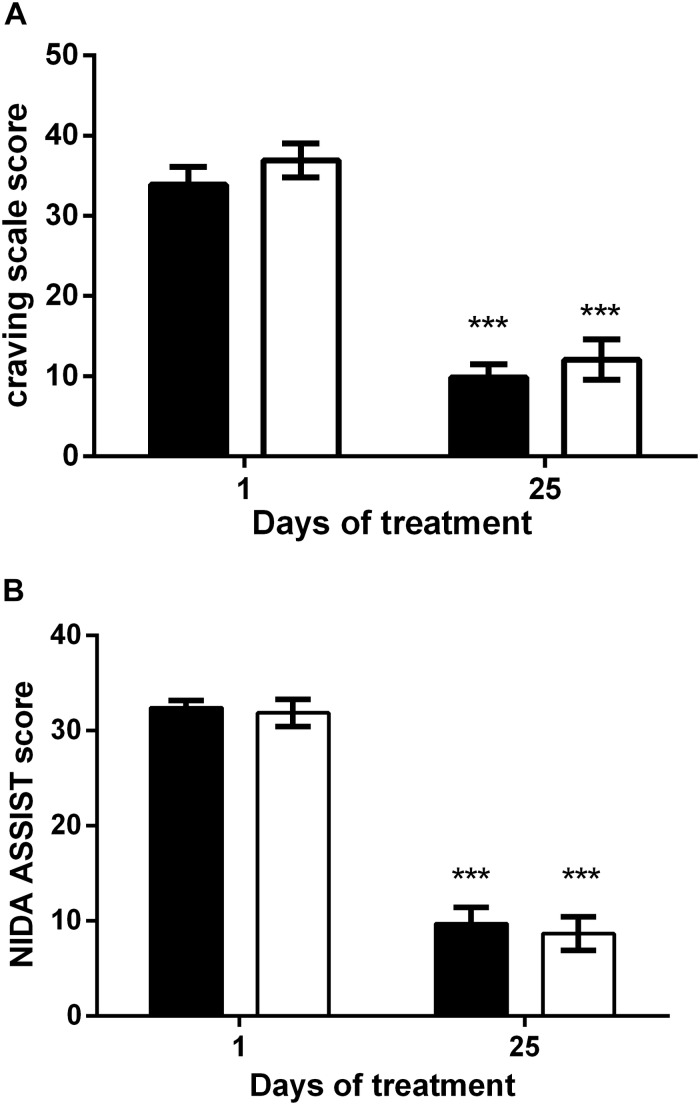
Reduction of the craving scale **(A)** and WHO ASSIST scale **(B)** after HF (black columns) and iTBS (white columns) treatment. Data are presented as mean ± SEM. ^∗∗∗^*P* < 0.001 vs. Day 1.

Figure 3A shows the craving score in the HF (black bars) and the iTBS (white bars) groups. With respect to the baseline value, both treatments significantly reduce craving for cocaine with no statistical difference. Two-way ANOVA revealed a significant effect of time (*F*_1,90_ = 127.3; *P* < 0.0001) but not of treatment (*F*_1,90_ = 1.48) or time × treatment interaction (*F*_1,90_ = 0.03). In line with it, the reduction of the WHO ASSIST questionnaire score was similar for both protocols (Figure 3B), as two-way ANOVA revealed a significant effect of time (*F*_1,90_ = 232.9; *P* < 0.0001) but not of treatment (*F*_1,90_ = 0.27) or time × treatment interaction (*F*_1,90_ = 0.03).

## Discussion

The main finding of the present study is the efficacy of PFC-iTBS in reducing cocaine intake and craving in treatment-seeking CUD patients. In addition, our iTBS protocol is as safe and effective as a high frequency (15 Hz) protocol. In both HF and iTBS protocols, about 80% of urine screen test were negative for cocaine within the 4th week of treatment and the majority (72 and 75%, respectively) of the patients reported to have quitted cocaine consumption by the same time. In line with it, craving significantly decreased by the end of treatment. We did not find significant differences between the two protocols for any of the parameter analyzed, showing that iTBS is as efficacious as HF stimulation in reducing cocaine intake. Dropouts and adverse side effects were also similar in the two protocols. Importantly, in our patients we did not observe focal seizures or any other transient neurological event that have been occasionally reported for both rTMS (Hu et al., 2011) and, more recently, for iTBS (Steele et al., 2018).

At present, only a limited number of studies have been conducted to explore the potential therapeutic effect of TMS in treating cocaine addiction (for recent comprehensive reviews see Bolloni et al., 2018; Rachid, 2018). Previous studies aimed at treating cocaine-dependent patients used high frequency protocols ranging from 10 to 20 Hz, from 90 to 100% resting motor threshold (rMT), and from 600 to 2400 pulses during 1 up to 10 sessions lasting about half an hour (Camprodon et al., 2007; Politi et al., 2008; Terraneo et al., 2016). We previously described that bilateral rTMS of PFC at 10 Hz reduces cocaine intake in CUD patients (Bolloni et al., 2016), an effect still present after 6 months. Intermittent TBS is a safe and effective procedure in the treatment of neurological disorders, such as Parkinson’s disease (Suppa et al., 2016), and psychiatric disorders, such as Tourette’s syndrome, schizophrenia and obsessive-compulsive disorder (Rachid, 2017). Pilot studies showed beneficial effects of iTBS in both addicted (Dieler et al., 2014) and depressed (Blumberger et al., 2018) patients but no study so far has tested its effectiveness in cocaine-dependent patients. To this regard, our study is the first to show that iTBS is safe and effective also in reducing cocaine intake and craving in addicted patients and that its effects are not inferior to those obtained by using conventional HF rTMS protocols. However, a clarification between effects on craving and intake deserves attention. Craving is a core symptom of addictive disorders and refers to the strong sense of compulsion to take the drug again, either to diminish negative effects or re-experience its positive effects. Although frequently studied, craving cannot, however, be interpreted as a ‘proxy’ of cocaine intake, which remains the main desirable clinical outcome. A number of compounds, such as topiramate, disulfiram, or N-acetylcysteine, are known to diminish the desire to use cocaine and, hence, to induce anti-craving effects (for a review see Lin, 2014). Yet, their anti-craving effects not always parallel their effects on drug intake, which represent an important clinical problem since reduction in drug consumption is clinically much more desirable than reduction in craving, i.e., the most critical achievement. Therefore, the efficacy of iTBS and high frequency (15 Hz) stimulation not only on craving but also on the intake of cocaine possesses clinical relevance.

In this study, iTBS targeted the PFC, a brain region regulating executive functions, levels of stress, motivation and reward, which are all strongly affected by exposure to drugs, including cocaine (Diana, 2013; Fattore and Diana, 2016; Diana et al., 2017). Chronic cocaine users typically exhibit decreased basal levels of dopamine receptors (Volkow et al., 1993) in the ventral and dorsal striatum and lower drug-induced dopamine release than non-addicted users, which implies a reduced experience of reward (Diana, 2011). During abstinence, characterized by dysphoria, anxiety and increased sensitivity to stress, craving is high and drug cues may easily trigger relapse. Motivation to seek the drug and inability to stop use are typically associated with changes in prefrontal circuits (involved in salience and attribution) and in hippocampus and amygdala (which mediate conditioned responses). After repeated exposure to cocaine the PFC, that in a drug-naïve conditions allows inhibitory control and promotes awareness of long-term consequences of emotional choices, becomes hypofunctional while limbic circuits are hyperactive, a combination than renders particularly difficult to stop the impulse of taking the drug. Since rescuing cocaine-induced hypoactivity in the PFC prevents compulsive drug seeking behavior (Chen et al., 2013), it seems reasonable to stimulate this area in an attempt to restore neural homeostasis by boosting dopamine signaling (Diana, 2011). In line with this, imaging studies revealed that high frequency rTMS targeting the PFC is able to increase the release of dopamine not only in the ventral striatum and cortical areas but also in the hippocampus, which is crucially involved in memory and engram retrieval (Strafella et al., 2001; Cho and Strafella, 2009; Diana et al., 2017). Along with the PFC, the insular cortex has also been tested as a promising target of TMS in drug addiction. Due to its projections to the PFC, the amygdala and the ventral striatum, the insula is in fact able to influence both the executive functions and reward-related behavior (Belin-Rauscent et al., 2016), and is activated during craving in cocaine addicts (Bonson et al., 2002). In light of these and other neurobiological and clinical evidence we decided to use in our study an H4 coil that was specifically designed to stimulate the PFC bilaterally and the insula symmetrically (Tendler et al., 2016).

Theta rhythms are present in many brain areas, including those regulating executive functions, which synchronize with related regions. Theta synchronized activity is considered a central neuronal mechanism supporting several cognitive functions as it subserves critical cognitive functions underlying the selection of a choice during goal-directed behavior (Womelsdorf et al., 2010). Rhythmic theta synchronization of neural circuits have been described in the evaluation of stimulus-reward associations and a significant relationship between theta oscillations and the reward system has been reported (Knyazev and Slobodskoy-Plusnin, 2009). Importantly, repeated use of cocaine can cause changes in synchronized network activity along limbic cortico-striatal circuits that may last long into abstinence (McCracken and Grace, 2013). Although with no data at hand at present, we could therefore speculate that iTBS in CUD patients may restore the synchronized network activity altered by chronic cocaine. PFC theta rhythms, for example, synchronizes with strongly connected structures like the anterior cingulate cortex and the posterior parietal cortex (Phillips et al., 2014; Babapoor-Farrokhran et al., 2017), which are both involved in regulating neuropsychological functions that became dysfunctional by cocaine use (Crunelle et al., 2012).

Although the results of this study are promising, we need to consider some caveats, including the small samples of patients involved in the study and the lack of a sham/control group, which does not control for placebo response. Moreover, certain aspects were not systematically assessed, including the psychiatric traits of the patients and the concomitant use of other drugs of abuse like alcohol, nicotine and cannabis. Moreover, further studies with larger samples of patients are needed to better assess the inter- and intra-individual variability in the efficacy of iTBS (Hinder et al., 2014) which is likely dependent upon the pre-intervention network connectivity of the stimulated cortical system (Nettekoven et al., 2015). Furthermore, other baseline stimulation parameters, such as intracortical inhibition and resting motor threshold, may contribute to the individual response pattern (López-Alonso et al., 2014).

## Conclusion

In conclusion, despite these limitations, this study shows the beneficial effects of iTBS in attenuating craving for cocaine, reducing intake and prolonging abstinence in treatment-seekers. Importantly, we show that iTBS and deep high-frequency rTMS are similarly effective. Considering that a typical iTBS treatment session (including setup) requires not more than 10 min, compared to the 25–30 min for standard 15 Hz rTMS, this relatively new protocol deserves further studies and clinical evaluations as it results more tolerable by patients by being less disruptive and less time-consuming than standard HF protocols.

## Data Availability

All datasets generated for this study are included in the manuscript and/or the supplementary files.

## Ethics Statement

The experimental protocol was approved by internal review board at the University of Sassari.

## Author Contributions

PB, GC, and MD conceived the study and designed the experiments. MD supervised the research. AS and VC performed the study. AS and LF analyzed the data. AS, LF, and MD discussed the data and prepared the manuscript. All authors read and approved the final manuscript.

## Conflict of Interest Statement

The authors declare that the research was conducted in the absence of any commercial or financial relationships that could be construed as a potential conflict of interest.
